# Analysis of the geographic distribution of HFRS in Liaoning Province between 2000 and 2005

**DOI:** 10.1186/1471-2458-7-207

**Published:** 2007-08-15

**Authors:** Hualiang Lin, Qiyong Liu, Junqiao Guo, Jibo Zhang, Jinfeng Wang, Huaxin Chen

**Affiliations:** 1 National Institute for Communicable Disease Control and Prevention, Chinese Center for Disease Control and Prevention, Beijing, China; 2 Liaoning Provincial Center for Disease Control and Prevention, Shenyang, China; 3 State Key Laboratory of Resources & Environmental Information System, Institute of Geographic Sciences and Natural Resources Research, Chinese Academy of Sciences, Beijing, China

## Abstract

**Background:**

Hemorrhagic fever with renal syndrome (HFRS) is endemic in Liaoning Province, China, and this province was the most serious area affected by HFRS during 2004 to 2005. In this study, we conducted a spatial analysis of HFRS cases with the objective to determine the distribution of HFRS cases and to identify key areas for future public health planning and resource allocation in Liaoning Province.

**Methods:**

The annual average incidence at the county level was calculated using HFRS cases reported between 2000 and 2005 in Liaoning Province. GIS-based spatial analyses were conducted to detect spatial distribution and clustering of HFRS incidence at the county level, and the difference of relative humidity and forestation between the cluster areas and non-cluster areas was analyzed.

**Results:**

Spatial distribution of HFRS cases in Liaoning Province from 2000 to 2005 was mapped at the county level to show crude incidence, excess hazard, and spatial smoothed incidence. Spatial cluster analysis suggested 16 and 41 counties were at increased risk for HFRS (p < 0.01) with the maximum spatial cluster sizes at ≤ 50% and ≤ 30% of the total population, respectively, and the analysis showed relative humidity and forestation in the cluster areas were significantly higher than in other areas.

**Conclusion:**

Some clustering of HFRS cases in Liaoning Province may be etiologically linked. There was strong evidence some HFRS cases in Liaoning Province formed clusters, but the mechanism underlying it remains unknown. In this study we found the clustering was consistent with the relative humidity and amount of forestation, and showed data indicating there may be some significant relationships.

## Background

HFRS is a zoonosis caused by different species of hantavirus (HV) causing fever, hemorrhage, kidney damage and hypotension. HFRS in China has the most severe epidemics having about 90% of the total reported HFRS cases in the world [1-4]. Although some prevention and control measures such as scientific rodent control, vaccination and environment management have been performed, HFRS remains a serious public health problem with about 20,000~50,000 human cases annually in mainland China [5]. And Liaoning Province is one of the most serious affected areas with the most cases in China and the highest incidence during the years 2004 and 2005. The incidence of HFRS shows high variability at the county level. The environmental factors, climatic factors, low social and economic development, and lack of health consciousness are thought to be related to the high incidence and spatial distribution of HFRS [3,4]. A better understanding of the spatial distribution patterns of HFRS and the relationship between the high incidence and relevant factors would help to identify areas and population at high risk and may better prevent and control HFRS.

Spatial analysis with spatial smoothing and cluster analysis are commonly used to characterize spatial patterns of disease [6-10]. Spatial smoothing is used to reduce random variation associated with small populations and enables observation of gradients or holes in disease incidence that may not be apparent from direct observation of the raw data. Spatial cluster analysis is conducted to identify whether cases of disease are geographically clustered [11-13]. In this study, we conducted GIS-based spatial analysis involving spatial smoothing and spatial clustering analysis to characterize geographic distribution patterns of HFRS cases. Spatial analysis was used to identify the distribution pattern of HFRS and population at high risk at the county level. The technique corrects for multiple comparisons, adjusts for the heterogeneous population densities among the different areas, detects foci without prior specification of suspected location or size, and thereby overcomes pre-selection bias and allows for adjustment of confounders [13,14].

## Methods

### Data collection and management

The study site is located in Liaoning Province, one of the three northeastern provinces of China (118°53'~125°46', 38°43'~43°26'N). The maximum distance from east to west is about 550km and from north to south is also about 550km. The area has a population of 41,940,051 (fifth national census in 2000) and encompasses 145,900 square kilometers.

There are 19 counties, 8 autonomic counties, 17 county-level cities, and 14 cities in Liaoning Province. For our study, we considered the city-governed region and others as county level, therefore there are 58 county-level study areas. The information includes the number of HFRS cases per month and per year in every county from 2000 to 2005. For the 6-year period (2000~2005), the average annual incidence was 8.65 cases per 100,000 persons, 21,760 cases were involved in this study.

HFRS cases were first diagnosed using clinical symptoms, then blood samples were collected in the hospitals, serologic identification was performed at the laboratory of Liaoning Provincial Center for Disease Control and Prevention (CDC) to confirm the clinical diagnosis, and then the data was collected by case number according to the sampling results. There might be admission rate bias in the disease report, but this has been reduced as much as possible.

### GIS mapping and smoothing for the incidence of HFRS

Records on HFRS cases between 2000 and 2005 were obtained from Liaoning Provincial CDC. To conduct a GIS-based analysis on the spatial distribution of HFRS, a county-level polygon map at a scale of 1:1,000,000 was obtained and on which the county-level point layers containing information regarding latitudes and longitudes of central points of each county were created. Demographic information based on the fifth national census (2000) was integrated in terms of an administration code [17]. All HFRS cases were geocoded and matched to the county-level layers on the polygons and points by administration code using the software ArcGIS9.1.

Based on annual average incidence, all counties were grouped into four categories: non-endemic areas, low endemic areas with an annual average incidence between 0 and 5 per 100,000, medium endemic areas with an incidence between 5 and 30 per 100,000, and high endemic areas with an incidence over 30 per 100,000. The four types of counties were color-coded on the maps.

To reduce variations of incidence in areas with a small population, annual average incidence of HFRS per 100,000 for each county over the 6-year period were calculated and spatial smoothing was used.

### Spatial cluster analysis

A "cluster" used here is a real or perceived aggregation of more than the expected number of HFRS cases in a population over a specified time period. In the present study, spatial cluster analysis was also performed on the confirmed HFRS cases to test whether the cases were distributed randomly over space and time, and if not, to evaluate any identified spatial and temporal disease clusters for statistical significance. A spatial scan statistic (SaTScan) [15] was then applied to identify clusters of HFRS. The spatial scan statistic places a circular window of varying size on the map surface and allows its center to move so that at any given position and size, the window includes different sets of adjacent neighborhoods. If the window contains the center of a neighborhood, then the whole neighborhood is included in the window. As the window is placed at each neighborhood center, its radius is varied continuously from zero up to a maximum radius which never includes more than 50% of the total population. The method creates a large number of distinct circular windows, each containing a distinct set of adjacent neighborhoods, and each is a possible candidate for containing a cluster of HFRS cases. For each window, the method uses a Monte Carlo simulation to test the null hypothesis that there is not an elevated risk of HFRS. Details of how the likelihood function is maximized over all windows under the Poisson assumption have been described elsewhere [12,13,15]. In this study, retrospective spatial cluster analysis for higher incidence was used, in which the maximum window radius was set to be smaller than 50% and 30% of the total population, respectively, to find possible sub-clusters. For each window of movable position and size change, the software tested the risk of HFRS within and outside the window using the null hypothesis of the same risk.

### The statistical analysis of relative humidity and forestation

Data analysis was conducted using the Statistical Package for the Social Sciences (SPSS). T-test analysis was conducted on the average relative humidity over the six-year period and forestation in every county between the cluster areas and the other areas.

## Results

### Spatial distribution of HFRS in Liaoning Province

**Figure 1:**
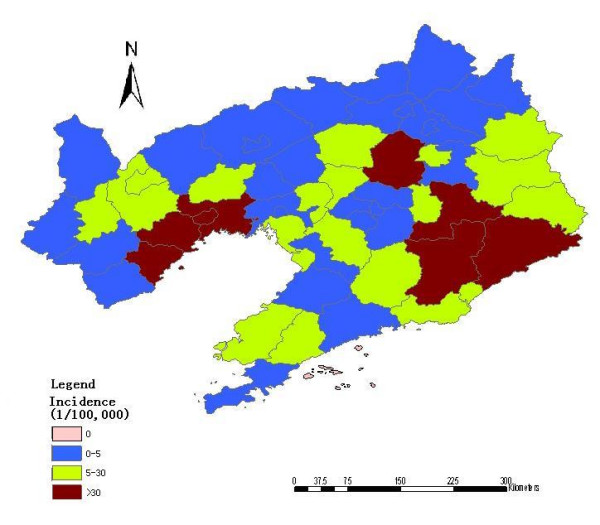
Distribution of HFRS in Liaoning Province, China, 2000–2005.

There were a total of 21,760 HFRS cases reported in Liaoning Province, China, from 2000 to 2005. The annual average incidence at the county-level ranged from 0 to 141.69 per 100,000. Among the 58 counties in Liaoning Province, only one county was non-endemic (covering 0.03% of the total land and 2.30% of the total population), 29 counties were low-endemic (covering 48.41% of the total land and 63.81% of the total population), 20 counties were medium-endemic (covering 33.27% of the total land and 26.77% of the total population), and eight counties were high-endemic (covering 18.29% of the total land and 7.13% of the total population). The areas of the four types are displayed in a thematic map as shown in Figure 1.

**Figure 2:**
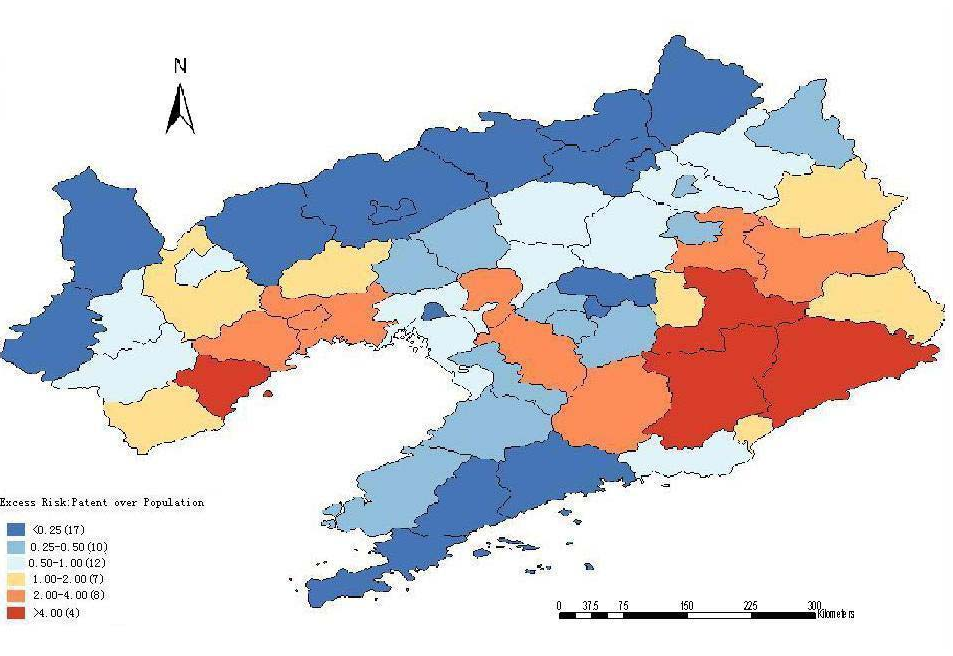
Excess risk map of HFRS in Liaoning Province, China, 2000–2005.

The excess hazard map (Figure 2) shows distribution of excess risk which was defined as a ratio of the observed number over the expected number of cases. Counties in blue color had lower risk than expected as indicated by excess risk of values of less than one. In contrast, counties in red color had higher risk than expected or excess risk values greater than one (Figure 2). The excess risk is a non-spatial analysis which ignores the influence of spatial autocorrelation.

**Figure 3:**
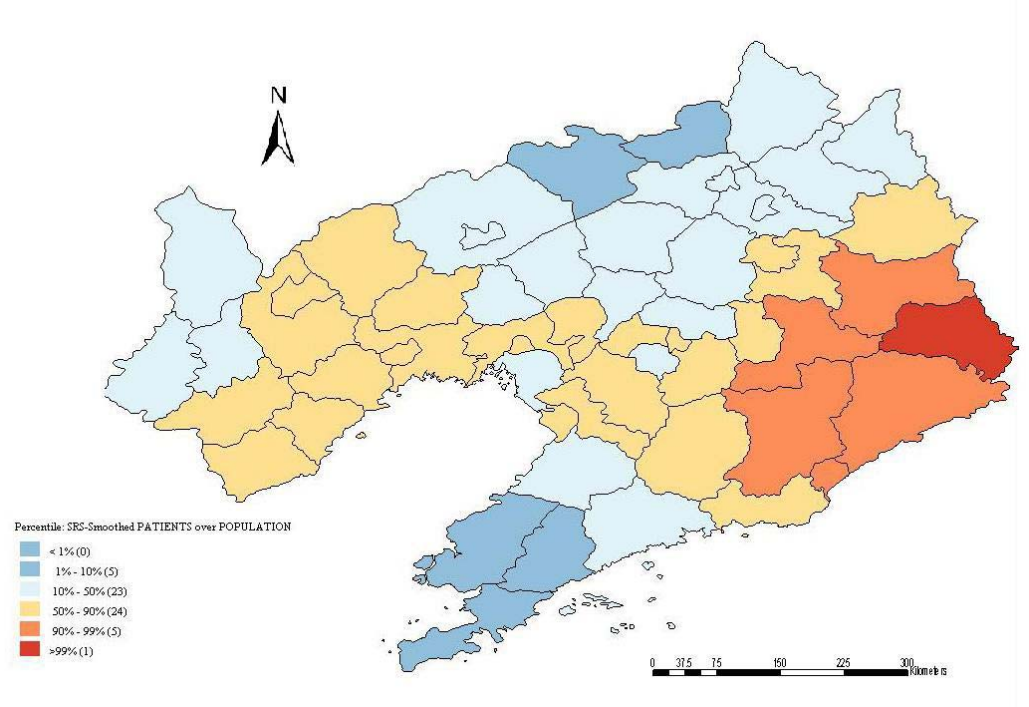
Spatial smoothed percentile map of HFRS in Liaoning Province, China, 2000–2005.

A spatially smoothed percentile map for annual average incidence was created by correcting the variance in the variability of incidence, and six neighbors identified for each county by k-nearest neighbor criterion provided the most appropriate map of smoothed incidence (Figure 3).

### Spatial clustering of HFRS in Liaoning Province

**Figure 4:**
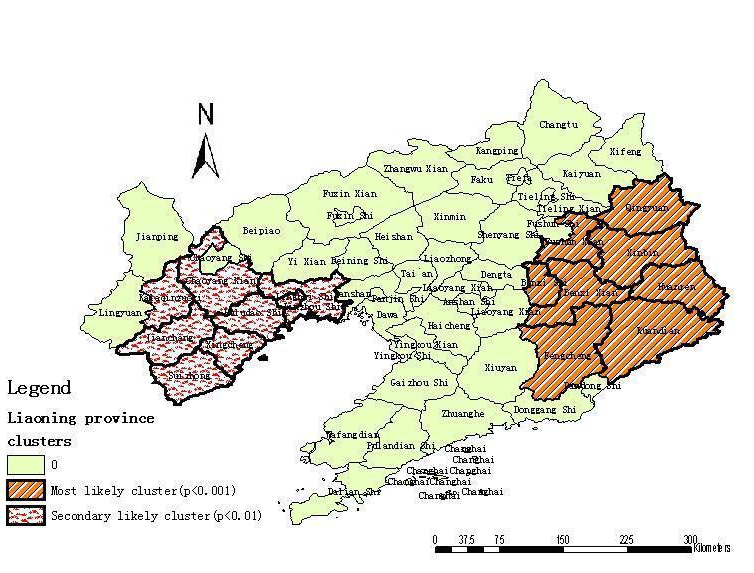
Spatial distribution of clusters of HFRS with significant higher incidence using the maximum cluster size < 50% of the total population in Liaoning Province, China, 2000–2005.

Analysis of cases of HFRS in 2000–2005 in a 58-county area in Liaoning Province, China, showed HFRS was not distributed randomly. Using the maximum spatial cluster size of ≤ 50% of the total population, a most likely cluster and a secondary cluster were identified (Figure 4). The most likely cluster encompassed eight counties where 8.04% of the total population resides. The overall RR within the cluster was 5.121 (p = 0.001) with an observed number of cases of 3,841 compared with a calculated 874.29 expected cases. The secondary cluster also included eight counties with 12.15% of the total population where the relative risk was 3.193 (p = 0.001) (Table 1).

**Table 1:** SaTScan statistics for the most likely cluster, Liaoning Province, China, 2000–2005. (Total population = 41,940,051 Total cases =21,760)

SaTScan statistics		Most likely cluster	Secondary clusters			
			
			2	3	4	5
The 1^st ^iteration^1^	Observed^a^	3841	3258			
	Expected^b^	874	1137			
	LLR^c^	2940	1421			
	RR^d^	5.121	3.193			
	p-value	0.001	0.01			
The 2^nd ^iteration^2^	Observed^a^	2697	2623	142	499	274
	Expected^b^	510	820	25	323	168
	LLR^c^	2423	1326	130	42	28
	RR^d^	5.898	3.499	5.749	1.556	1.634
	p-value	0.001	0.001	0.001	0.01	0.01

a Observed: The number of observed cases in a cluster.

b Expected: The number of expected cases in a cluster.

c LLR: Log likelihood ratio.

d RR: Relative risk

1 Maximum cluster size set at 50% of the study population.

2 Maximum cluster size set at 30% of the study population.

**Figure 5:**
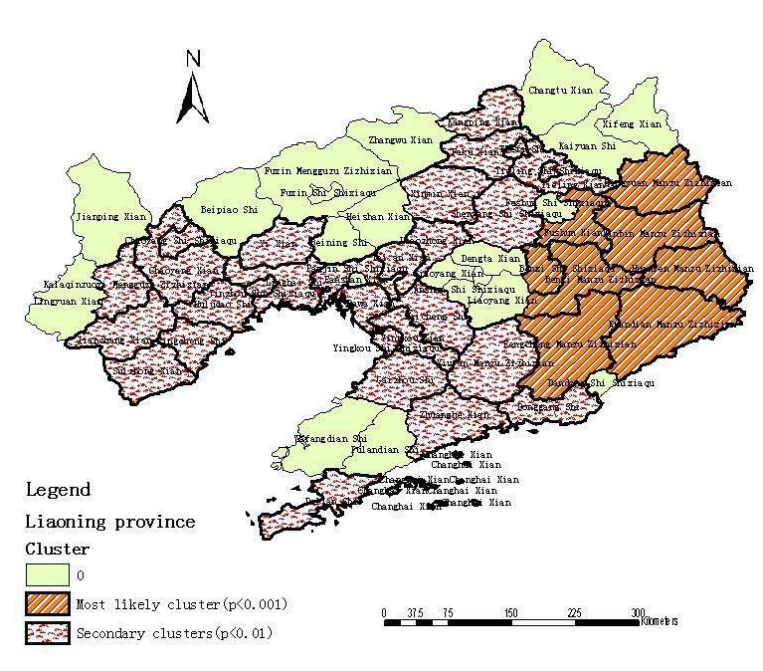
Spatial distribution of clusters of HFRS with significant higher incidence using the maximum cluster size < 30% of the total population in Liaoning Province, China, 2000–2005.

To investigate the possibility of smaller clusters, the same analysis was performed with a modification of the maximum spatial cluster size which was defined as ≤ 30% total population. A most likely cluster and four secondary clusters were identified (Figure 5). The most likely cluster was the same as in the 50% analysis. Four secondary sub-clusters included 29 counties which contain 61.50% of the total population. This excess risk within a nonrandom distribution pattern was also significant (p < 0.01) (Table 1).

### The incidence of HFRS compared to relative humidity and forestation

**Figure 6:**
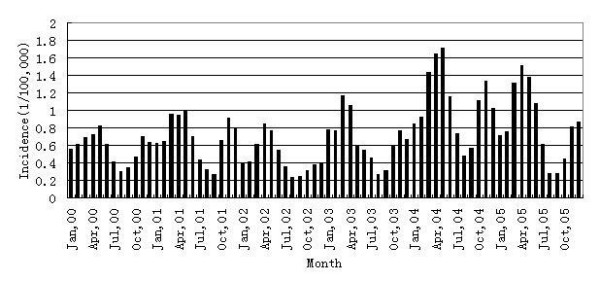
Monthly incidence of HFRS in Liaoning Province, China, 2000–2005.

There were two epidemic peaks almost in every year during the period 2000–2005 in Liaoning Province. One was in March to May, and the other in October to January (Figure 6). There was a clear seasonal trend in the epidemic of HFRS in Liaoning Province. We saw the largest incidence was in March to May in the year 2004.

The difference in relative humidity and forestation between the cluster areas and non-cluster areas was analyzed and the results are shown in Table 2. The differences were all significant between the first cluster areas and the others, while some in the second cluster areas and non-cluster areas were not significant, and gave us some indications the relative humidity and forestation may have some contribution to the high epidemic occurrence of HFRS in Liaoning Province.

**Table 2:** The comparison of relative humidity and forestation between cluster areas and non-cluster areas

Variables	1^st ^cluster and others		2^nd ^cluster and non-cluser	
	
	t-value	p-value	t-value	p-value
Forest amount	3.32^a^	0.01^a^	2.07^a^	0.04^a^
	3.32^b^	0.01^b^	1.14^b^	0.26^b^
Relative humidity	2.44^a^	0.02^a^	2.05^a^	0.046^a^
	2.44^b^	0.02^b^	0.87^b^	0.39^b^

a Maximum cluster size set at 50% of the study population.

b Maximum cluster size set at 30% of the study population.

## Discussion

Spatial variation in disease distribution occurs where multiple statistical methods have been developed to determine whether patterns of variation occur by chance alone, or whether variation is unlikely to have happened at random [16-18]. Using GIS-based spatial statistics, we investigated the spatial distribution of HFRS cases and identified counties with high endemic HFRS and clustering patterns. The Martin Kulldorff's spatial scan test has several advantages over other local cluster detection tests, namely, a formal likelihood test statistic, avoidance of multiple testing, and not restricting scanning for clusters of a pre-specified size, thereby avoiding pre-selection bias [19]. The spatial statistics analysis yields a nonrandom distribution of HFRS within the 58-county area. We identified the eastern counties as having a cluster in Liaoning Province which was not previously reported. The relative risk in this cluster was at the highest level of 5.121 (50% analysis) and 5.898 (30% analysis). Although much is still unknown about HFRS epidemic initiation and development, there is sufficient data presented here to argue that HFRS is most likely influenced by a complex combination of factors rather than a single foci pathogenic factor. It is likely that many factors contribute to its epidemic and development from natural environmental agents to economic and social factors.

Liaoning Province can be divided into three parts in respect to terrain: the Liaodong mountainous region, the Liaohe plain region, and the Liaoxi mountainous region [20]. We found as shown in Figure 4 the relatively higher risk areas are in the eastern and western mountainous regions where these regions have a humid or semi-humid climate with an annual average precipitation of 650–1,200 mm and 500 – 600 mm, respectively [20]. There is more forest in these regions than on the Liaohe plain, which contribute to a habitat for rodents, the reservoir and vector of HV, and increased opportunities of human contact with rodents and their excreta. Therefore, the terrain, the amount of forestation and the relative high humidity may be the important factors responsible for the epidemic development of HFRS in Liaoning Province.

As mapping and spatial analysis software become easier to use, public health professionals can increasingly employ these tools to aid in disease surveillance and cluster investigations [21]. The spatial scan statistic method approach illustrated here is a useful instrument for HFRS analysis of cluster patterns. Further, space-time disease-surveillance methods have been proposed as a dynamic supplement to purely spatial statistical methods for outbreak detection to detect and predict localized outbreaks before they spread to larger regions. Therefore, this study should be used as a preliminary study showing only spatial patterns. In the areas in which HFRS are highly epidemic, aiming prevention strategies at areas of highest risk can potentially increase the health maintenance program's effectiveness. Persons at highest risk should be informed of the risks and methods for risk reduction. Funds spent on these programs may be better spent in the areas identified here where cost-effectiveness can be maximized. The data presented here will assist state and local health departments for HFRS surveillance systems in the investigation of HFRS clusters by providing definitions and recommendations to aid in the development of a better prevention strategy.

## Conclusion

This study analyzed the spatial distribution of HFRS in Liaoning Province, China, during 2000 to 2005 using the spatial smoothing and spatial scan statistic method. SaTScan identified a geographic area in eastern Liaoning Province as the most likely endemic cluster. This has not been previously reported. The data provide evidence there are environment factors including the amount of forestation and climatic factors that may contribute to cause these clusters. The data show practical HFRS control measures, as well as methods for future study of HFRS and other vector-borne diseases. Further, GIS and GIS-based spatial statistical techniques may provide an opportunity to clarify and quantify the epidemic situation of HFRS within highly epidemic areas, and lay a foundation to pursue future investigations into the environmental factors responsible for the increased disease risk. To implement specific and geographically appropriate risk-reduction programs, the use of such spatial analysis tools should become an integral component in epidemiology research and risk assessment of HFRS.

## Competing interests

The author(s) declare that they have no competing interests.

## Authors' contributions

HL, JZ and HC were involved in the conceptualization, execution, and writing of the first draft of this manuscript. HL, JW, QL and JG contributed to the research design. All authors were involved in the preparation of the manuscript.

## Pre-publication history

The pre-publication history for this paper can be accessed here:


